# Mitotic Defects Lead to Pervasive Aneuploidy and Accompany Loss of RB1 Activity in Mouse *Lmna^Dhe^* Dermal Fibroblasts

**DOI:** 10.1371/journal.pone.0018065

**Published:** 2011-03-25

**Authors:** C. Herbert Pratt, Michelle Curtain, Leah Rae Donahue, Lindsay S. Shopland

**Affiliations:** The Jackson Laboratory, Bar Harbor, Maine, United States of America; Instituto Nacional de Câncer, Brazil

## Abstract

**Background:**

Lamin A (*LMNA*) is a component of the nuclear lamina and is mutated in several human diseases, including Emery-Dreifuss muscular dystrophy (EDMD; OMIM ID# 181350) and the premature aging syndrome Hutchinson-Gilford progeria syndrome (HGPS; OMIM ID# 176670). Cells from progeria patients exhibit cell cycle defects in both interphase and mitosis. Mouse models with loss of LMNA function have reduced Retinoblastoma protein (RB1) activity, leading to aberrant cell cycle control in interphase, but how mitosis is affected by LMNA is not well understood.

**Results:**

We examined the cell cycle and structural phenotypes of cells from mice with the *Lmna* allele, Disheveled hair and ears (*Lmna^Dhe^*). We found that dermal fibroblasts from heterozygous *Lmna^Dhe^* (*Lmna^Dhe/+^*) mice exhibit many phenotypes of human laminopathy cells. These include severe perturbations to the nuclear shape and lamina, increased DNA damage, and slow growth rates due to mitotic delay. Interestingly, *Lmna^Dhe/+^* fibroblasts also had reduced levels of hypophosphorylated RB1 and the non-SMC condensin II-subunit D3 (NCAP-D3), a mitosis specific centromere condensin subunit that depends on RB1 activity. Mitotic check point control by mitotic arrest deficient-like 1 (MAD2L1) also was perturbed in *Lmna^Dhe^* ^/+^ cells. *Lmna^Dhe^* ^/+^ fibroblasts were consistently aneuploid and had higher levels of micronuclei and anaphase bridges than normal fibroblasts, consistent with chromosome segregation defects.

**Conclusions:**

These data indicate that RB1 may be a key regulator of cellular phenotype in laminopathy-related cells, and suggest that the effects of LMNA on RB1 include both interphase and mitotic cell cycle control.

## Introduction

The nuclear lamina is a network of Type V intermediate filament proteins that lie primarily beneath the inner nuclear membrane. In mice and humans, it is composed of both A-type (A, AΔ10, C and C2) and B-type (B1 and B2) lamins. A-type lamins are generated by alternative splicing of one gene (*Lmna* in mice, *LMNA* in humans) and B-type lamins are encoded by two genes, *Lmnb1*/*LMNB1* and *Lmnb2/LMNB2* [1]–[3]. The Lamin subtypes form independent, but interconnected, lattice-like meshworks that provide structural stability to the nucleus [4]. The nuclear lamina also participates in cell cycle control, DNA replication, gene transcription, genome three-dimensional (3-D) architecture and stabilization of the nucleus in the cytoplasm [1]–[7]. Perturbations to *LMNA* have dire effects on the cellular processes, including the cell cycle. Although recent work has begun to describe the consequences of *LMNA* defects on the cell cycle, the mechanisms underlying these effects are poorly understood.

Over 250 disease mutations have been mapped to *LMNA*. These are associated with 12 disorders collectively called laminopathies, which affect a wide variety of cell types and tissues [1], [2], [8]–[10]. Mutations in *LMNA* manifest as such varied disorders as muscular dystrophies, lipodystrophies, dermopathies, cardiomyopathies, and progeria syndromes, including Hutchinson-Gilford progeria (HGPS; OMIM ID# 176670) [1], [2], [8]–[10]. Most cells expressing a mutated *LMNA* gene share a few common phenotypes, including nuclear membrane blebbing and delayed cell cycling, yet the molecular mechanisms governing these phenotypes are currently unclear.

Recent work has begun to make inroads to understanding cell cycle defects in *LMNA*/*Lmna* mutant cells [6], [11]–[16]. Cells from *Lmna* knock-out mice, as well as mice deficient for LMNA interacting proteins LAP2α and ZMPSTE-24, have defects in the G_1_/S-phase transition, due to reduced levels of hypophosphorylated retinoblastoma protein (RB1). Normal interactions among RB1 and a soluble, intranuclear pool of LMNA and LAP2α are disrupted in these mutant cells [11], [13], [14], [16]. Cells from human progeria and muscular dystrophy patients have gene expression signatures that implicate central defects in RB1 activity as well [16], [17]. However, for progeria cells, a direct link to RB1 signaling has yet to be demonstrated. Cell populations from progeria patients and mice with HGPS-related lamin A alleles do grow more slowly than normal cells. This is in part due to persistent DNA damage and telomere defects, which lead to increased cellular senescence [18]–[23]. In addition, cells expressing *LMNA^HGPS^* have aberrant mitotic progression and aneuploidy [6], [7], [12], [24].

At the start of mitosis the nuclear lamina must break down to allow for proper attachment of the chromatids to the mitotic spindle [25]. The checkpoints regulating chromosome spindle attachment, congression at the metaphase plate and separation into daughter cells at anaphase are highly regulated. Condensin II is a multi-subunit protein complex that condenses mitotic chromosomes prior anaphase. One subunit of this complex, the non-SMC condensin II-subunit D3 (NCAP-D3) functions at the centromeric regions of chromosomes and is required to maintain centromeric cohesion. Recent studies in primary cells and tumor cell lines have shown that decreased expression of RB1 causes decreased NCAP-D3 levels, which resulted in a more disorganized metaphase plate and chromosome missegregation [23], [26]–[28]. Thus, although RB1 is often thought to exert its influence at the G_1_/S-phase transition, perturbations to RB1 have consequences further downstream in the cell cycle, specifically during mitosis. These recent findings suggest that the perturbations to RB1 in both human and mouse lamin A mutant cells could manifest at both the G_1_/S-phase transition and in mitosis.

In this study, we examined features of the cell cycle using a newly described *Lmna* mouse model, Disheveled hair and ears (*Lmna^Dhe^*), which exhibits a subset of phenotypes of HGPS and other non-muscular dystrophy laminopathies, including epidermal dysplasia, craniofacial abnormalities, and markedly thinned hypodermal fat layers [5]–[7], [19], [29]. The *Lmna^Dhe^* allele is a spontaneous point mutation in the first coiled-coil domain of lamin A and C (L52R), suggesting it significantly perturbs lamina structure and function [29]. Indeed, we found that dermal fibroblasts from heterozygous *Lmna^Dhe^* mice (hereafter called *Lmna^Dhe/+^*) had nuclear morphology defects and increased DNA damage, as well as mitotic defects, including aneuploidy, lagging chromosomes and anaphase bridges. Our data indicate that these mitotic defects are associated with low levels of activated RB1 and CAPD3, as well as other defects in the mitotic spindle checkpoint, suggesting a new role for RB1 in the mitotic defects of laminopathies.

## Materials and Methods

### Mice

The B6(D2)-*Lmna^Dhe^*/TyJ mice arose on the C57BL/6J segment of the BXD8/TyJ strain, and the mutation has now been made congenic on the C57BL/6J inbred strain through repeated backcrosses (N50F1). The mice were maintained under 14∶10 hour light∶dark cycles; autoclaved diet NIH-31 (6% fat, 18% protein, Ca∶P 1∶1, vitamin and mineral fortified; PMI, Richmond, IN) and HCl acidified water (pH 2.8–3.2) were provided *ad libitum* [29]. Mice were housed in groups of 4 or 5 within polycarbonate boxes of 51 square inch area on sterilized shavings of Northern White Pine as bedding. All procedures were approved by The Jackson Laboratory's Institutional Animal Care and Use Committee and performed in accordance with National Institutes of Health guidelines for the care and use of animals in research (ACUC Policy # 99066).

### Genotyping

The following PCR primers flanking the *Lmna^Dhe^* mutation in exon 1 were used to amplify genomic DNA extracted from tail tips: dhefwd 5′- ACCTGCAGGAGCTCAATGAC-3′, dherev 5′ –TGAACTCCTCACGCACTTTG – 3′. PCR products then were digested with SmaI, which produces a 240 bp DNA fragment for the *Lmna^+/+^* allele and 186 bp and 54 bp DNA fragments for the *Lmna^Dhe^* allele [29].

### Cell Culture

Primary dermal fibroblast skin explant cultures were obtained using neonatal (8 day old) *Lmna^+/+^*and *Lmna^Dhe/+^* mice as previously described [30]. Briefly, we excised dorsal skin from just posterior to the occipital bone to just anterior to the tail base and from 1 mm dorsal to the limbs on either side. Any remaining subcutaneous fat and muscle was then trimmed and skin was washed twice in sterile, ice-cold 1× Phosphate Buffered Saline (PBS). Skin was cut into 2 mm×2 mm squares and washed in sterile, ice-cold 1× PBS. Skin explants were placed into 100 mm^2^ cell culture dishes dermal side down, covered with sterile, ethanol-washed glass coverslips and Dulbecco's Minimal Essential Media (DMEM; Gibco, Carlsbad, CA) supplemented with 10% Fetal Bovine Serum (FBS; Lonza, Walkersville, MD) and 2% antibiotic/antimycotic (Gibco, Carlsbad, CA). Plates were placed in a humidified cell culture incubator at 37°C with 5% CO_2_ and 2% O_2_. Media was replaced every 2 days with fresh media for 7 days until fibroblasts emerged from edges of skin explants. Fibroblasts were then passaged to additional coverslips for immunofluorescence or harvested for protein analysis.

### Immunofluorescence

Dermal fibroblasts were fixed for 10 minutes in 4% formaldehyde, 1× PBS and permeabilized for 10 minutes with 0.5% Triton X-100 in 1× PBS. Next, cells were immunostained with goat anti-LMNA 1∶50 (sc-6218, clone N-18), goat anti-LMNB 1∶200 (sc-6217, clone M20) (Santa Cruz Biotech, Santa Cruz, CA), rabbit anti-γH2Ax 1∶400 (Bethyl Laboratories, Montogomery, TX), rabbit anti-α-Tubulin 1∶50 (Abcam, Cambridge, MA), and/or rabbit anti-Ki-67 1∶150 (Novocastra, Leica Microsystems, Deerfield, IL) and detected with either Alexa Fluor 488–donkey anti–goat IgG 1∶200 (Invitrogen, Carlsbad, CA), Rhodamine-donkey anti-mouse IgG 1∶200 or Rhodamine-donkey anti-rabbit IgG 1∶200 (Jackson ImmunoResearch, West Grove, PA). Cells were counterstained with 1 µg/ml 4,6-diamidino-2-phenylindole (DAPI; Sigma-Aldrich, St. Louis, MO) and mounted in a solution of 1% phenylenediamine (Sigma-Aldrich, St. Louis, MO) in 1× PBS with 90% glycerol for imaging. 3-D images were acquired using either the Zeiss Axiovert 200M inverted microscope equipped with a Plan-Apochromat 100×/1.40 Oil objective (Carl Zeiss Microimaging, Inc., Thornwood, NJ) along with a Hamamatsu ORCA-ER digital camera (Hamamatsu Photonics, Bridgewater, NJ) or TCS SP5 confocal microscope (Leica Microsystems, Deerfield, IL) with a 100×1.4 NA oil objective lens. Three-dimensional (3D) image stacks were taken at 200 nm steps.

### Nuclear morphometry

3D wide-field epi-fluorescence images were deconvolved using AutoDeblur software (Media Cybernetics, Bethesda, MD). Anti-LMNB immunolabeled cell nuclei were scored for the presence or absence of one or more blebs, anaphase bridges and/or micronuclei in the nuclear lamina using cells from three mice from different litters for each genotype. Single optical sections through the middle of each cell nucleus were examined for blebbing, while all sections were examined for the presence of micronuclei and/or anaphase bridges. Blebs, micronuclei and anaphase bridges were scored independently for each cell counted. Therefore cells may exhibit either blebs, micronuclei, anaphase bridges or all three.

### Mitotic index

Cell fluorescently labeled with both anti-α-Tubulin and LMNA antibodies were directly scored using the Zeiss Axiovert 200M inverted microscope. Approximately 1000 cells from at least 3 different pups for both *Lmna^+/+^*and *Lmna^Dhe/+^* cultures were counted to determine the proportion of mitotic cells present. At least 100 mitotic cells from both *Lmna^+/+^*and mutant cultures also were scored as in prophase, prometaphase, metaphase, anaphase or telophase, based upon DAPI, *LMNA* and α-Tubulin labeling.

### Western Blot Analysis

Whole-cell protein extracts were prepared by trypsinizing cells and lysing in ice-cold extraction buffer (10 mM Imidazole, 100 mM NaCl, 1 mM MgCl_2_, 5 mM Na_2_EDTA and 1% Triton X-100) and homogenizing the cells using a pipette tip. The lysate was centrifuged at 14,000 g at 4°C for 15 minutes, after which the supernatant (Soluble Extract) was collected and protein concentration was determined using the Bradford assay [31]. The pellet (Insoluble Extract) was resuspended directly in 6× sample buffer (125 mM Tris-HCl pH 6.8, 2% Sodium dodecylsulfate (SDS), 20% Glycerol, 0.2% Bromophenol Blue and 5% β-Mercaptoethanol (BME)). Protein samples, 30 µg of soluble extract or cell number equivalent of insoluble pellet, were reduced in 6× sample buffer by boiling for 5 minutes and resolved on a 7.5% SDS-PAGE gel. Subsequently, the gels were electroblotted onto Polyvinylidene fluoride (PVDF) membranes (Invitrogen, Carlsbad, CA) at 115 mA for 9 hrs at room temperature. Non-specific binding of antibodies to the PVDF membrane was blocked by incubation in blocking buffer (5% nonfat dried milk in Tris-buffered saline with 0.1% Tween-20 (TBST)). Next, the blots were incubated with appropriate primary antibody overnight at 4°C diluted in blocking buffer as follows: goat anti-mouse *LMNA* 1∶1000 (sc-6215), goat anti-mouse LMNB 1∶1000 (sc-6217) (Santa Cruz Biotech, Santa Cruz, CA), rabbit anti-mouse Prelamin A 1∶2000 (Diatheva, Fano, Italy), mouse anti-mouse MAD2L1 1∶1000 (BD Transduction Labs, Lexington, KY), mouse anti-mouse NCAP-D3 1∶1000 (Millipore, Billerica, MA), mouse anti-mouse TRP53 1∶1000, rabbit anti-mouse RB1 1∶1000, and rabbit anti-mouse α-Tubulin 1∶5000 (Abcam, Cambridge, MA). This was followed by incubation of the blots for an additional hour in donkey anti-goat, donkey anti-mouse or donkey anti-rabbit IgG-HRP conjugate diluted to 1∶10,000 (Santa Cruz Biotechnology, Santa Cruz, CA) in blocking buffer. Signal detection was performed using the enhanced chemilluminescence-plus western blotting substrate (ECL+) (Pierce Biotechnology; Rockford, IL) followed by exposure to x-ray film.

### Static Adhesion Assay

50,000 dermal fibroblasts were seeded into each well of a 6-well cell culture plate. After either 3 hours (Day 0 time point) or 96 hours (Day 4 time point), media was removed and wells were rinsed three times with pre-warmed 1× PBS. Next , 500 µL of substrate solution (3.75 mM p-nitropheno-N-acetyl-β-D-glucosaminide (Sigma-Aldrich, St. Louis, MO), 0.1% Triton X-100, 50 mM citrate buffer, pH 5.0) was added to each well and the plates were incubated for 30 minutes at 37°C. After incubation, 750 µL of STOP solution (5 mM EDTA, 50 mM Glycine buffer, pH 10.4) was added to each well. Lastly, 100 µL of reaction mixture was loaded in triplicates into a 96-well plate and absorbance was measured on a microplate reader at 405 nm [32], [33].

### Senescence-associated β-galactosidase labeling (SA-β-gal)

Senescence was detected as previously described ([34]). Briefly, *Lmna^Dhe/+^* and *Lmna^+/+^*dermal fibroblasts were grown to 50% confluence. Cell cultures were then treated with 100 nM Bafilomycin A1 (Sigma-Aldrich, St. Louis, MO) for 1 hr to induce lysosmal alklynization. Cell cultures then were incubated with 33 µM 5-dodecanoylaminofluorescein di-ß-D-galactopyranoside (C_12_FDG) (Invitrogen, Carlsbad, CA) for 1 hr. After incubation cells were washed with pre-warmed 1× PBS, harvested by trypsinization and resuspended in ice-cold 1× PBS. Next, cells were suspended in propidium iodide working solution (0.1% Triton X-100, 10 mg/mL RNase A, 1 mg/mL Propidium Iodide (Sigma-Aldrich, St. Louis, MO)) for 15 minutes. Lastly, fluorescent cells were counted using a BD LSR II Flow Cytometer (BD Biosciences, San Jose, CA). The data were analyzed using FlowJo v.7 (Tree Star, Inc., Ashland, OR).

### Apoptosis assay

Annexin V labeling was performed as previously described [35], [36]. Briefly, fibroblasts were trypsinized, washed in 1× PBS and resuspended in 1× Annexin V Binding Buffer (BD Pharmingen, San Jose, CA) at a concentration of 1×10^6^ cells/mL. 1×10^5^ cells were removed and suspended in 5 µL PE-Annexin V (BD Pharmingen, San Jose, CA) and 0.2 µg/mL DAPI for 15 minutes at room temperature. Lastly, 400 µL of 1× Binding Buffer was added to each sample. Annexin V labeling was measured by flow cytometry as described above.

### DNA content measurements

For DNA content and cell cycle analysis, cells were trypsinized washed twice in 1× PBS, and fixed in ice-cold 70% ethanol for 2 hours at 4°C. Cells were washed twice with 1× PBS, resuspended in 200 µL of propidium iodide working solution (0.1% Triton X-100, 10 mg/mL RNase A, 1 mg/mL Propidium Iodide (Sigma-Aldrich, St. Louis, MO) for 15 minutes at 37°C, and analyzed by flow cytometry as described above [37].

### Spectral Karyotyping (SKY)

Metaphase spreads were prepared as previously described [38]. Metaphase slides were denatured in 70% Formamide (Sigma-Aldrich, St. Louis, MO) in 2× Saline-Sodium Citrate buffer (SSC) (Invitrogen, Carlsbad, CA) at 68°C for 30 seconds and dehydrated in a series of ethanol washes (70%, 80% and 100%). The mouse specific SKY paint (Applied Spectral Imaging, Migdal Ha'emek, Israel) was denatured in an 80°C water bath for 10 minutes and allowed to pre-anneal at 37°C for 15 minutes before being applied to chromosome preparations. Slides were hybridized overnight at 37°C in a humidified hybridization chamber. Slides were then washed in 4× SSC at 72°C and 4× SST (4×SSC/0.1% Tween) at 45°C for 5 minutes. The slides were incubated with CAD Antibody Detection Kits (Applied Spectral Imaging, Migdal Ha'emek, Israel) at 37°C for 45 minutes and washed in 4× SST at 45°C for 5 minutes before mounting in Vectashield (Vector Labs, Burlingame, CA) with DAPI. Slides were imaged and analyzed by an ASI SKY Workstation equipped with HiSKY software (Applied Spectral Imaging, Migdal Ha'emek, Israel). Automated karyotyping was followed by manual analysis to validate the karyotype.

## Results

### Aberrant nuclear lamina morphology in *Lmna^Dhe/+^* skin fibroblasts

**Figure 1:**
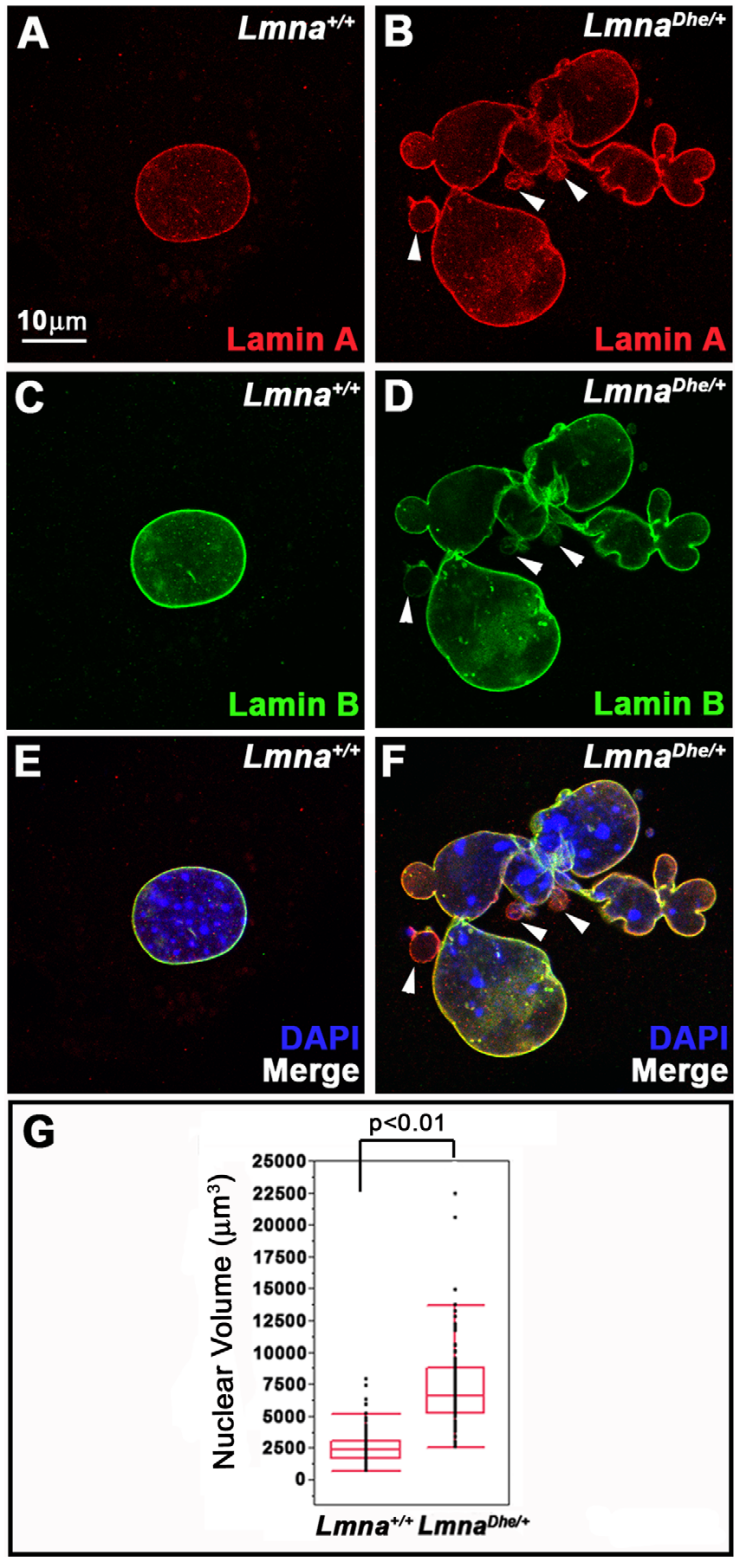
*Lmna^Dhe/+^* cells exhibit blebbed nuclear membranes and large nuclear volumes. (A–F) Immunodetection of Lamin A (red; A,B, E,F) and Lamin B (green; C–F) in *Lmna^+/+^*(A,C,E) and *Lmna^Dhe/+^* (B,D,F) fibroblasts reveals extensive blebbing of the nuclear envelope and exclusion of Lamin B from these blebs in mutant cells (white arrowheads in B,D,F). Single confocal optical sections through the middle of the nucleus, counterstained with DAPI (blue), are shown. (G) Box-and-whisker plot of nuclear volumes measured from 3D confocal image stacks indicates a significantly greater average volume and greater range in nuclear sizes in *Lmna^Dhe/+^* versus *Lmna^+/+^* fibroblasts (p<0.01; Student's t-test).

Previous work on *Lmna^Dhe/+^* mice had shown skin defects, cranial abnormalities, and extensive blebbing of the nuclear membrane in cranial osteoblasts [29]. To determine whether *Lmna^Dhe/+^* skin was similarly affected we cultured dermal fibroblasts from neonatal mice, then fixed and labeled them with anti-LMNA and -LMNB antibodies. Examination by wide-field fluorescence microscopy revealed large blebs and lobulations within *Lmna^Dhe/+^* fibroblasts nuclear membranes. These abnormal cells were significantly more frequent (41.78%+/−4.9%) in *Lmna^Dhe/+^* cultures than in *Lmna^+/+^*cultures (6.54%+/−1.4%) (p≤0.01; χ^2^-test) (Figure 1 A–F). In addition, while the LMNA signal was bright around the periphery of the entire nucleus of mutant cells, the LMNB signal was depleted in at least half of the blebbed regions (Figure 1B,D and F; white arrowheads). Many mutant nuclei appeared larger than *Lmna^+/+^* nuclei. 3-D confocal imaging and measurement of the nuclear volume confirmed that *Lmna^Dhe/+^* cells had a larger average nuclear volume than *Lmna^+/+^*counterparts (p≤0.001, Student's t-test). The range of nuclear volumes also was greater in *Lmna^Dhe/+^* cells, indicating large variations in nuclear size (Figure 1G).

**Figure 2:**
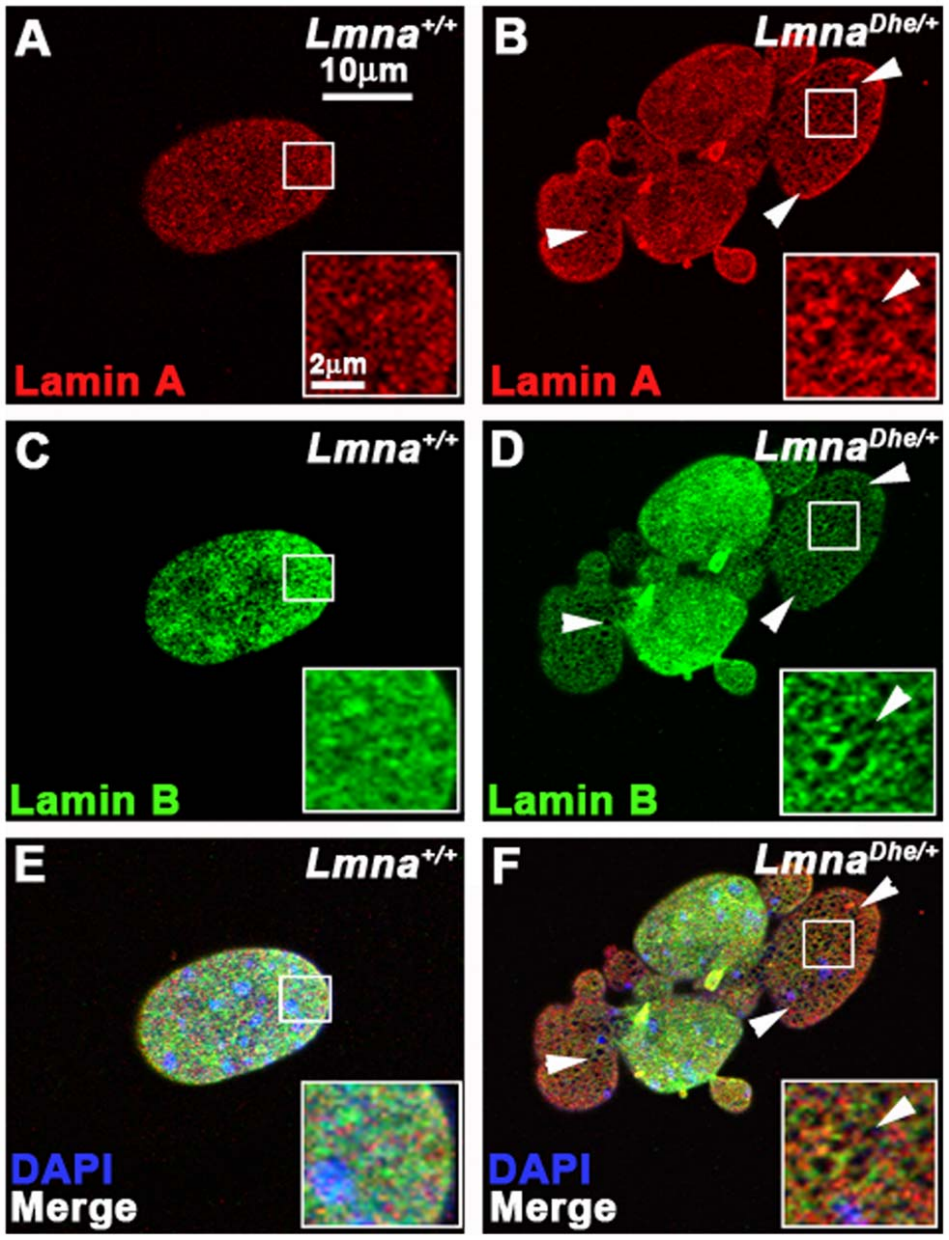
LMNA^Dhe^ affects both the Lamin A and Lamin B meshwork. (A–F) Immunodetection of Lamin A (red; A,B,E,F) and Lamin B (green;C–F) in *Lmna^+/+^*(A,C,E) and *Lmna^Dhe/+^* (B,D,F) fibroblasts reveals large, irregular holes in the Lamin A (white arrowheads in B) and Lamin B meshwork (white arrows in D) in mutant cells. Insets represent magnified portions of an area of the nucleus indicted by the white boxes. Single confocal optical sections through the top of the nucleus are shown. (E, F) Merged images with nuclei counterstained with DAPI (blue).

**Figure 3:**
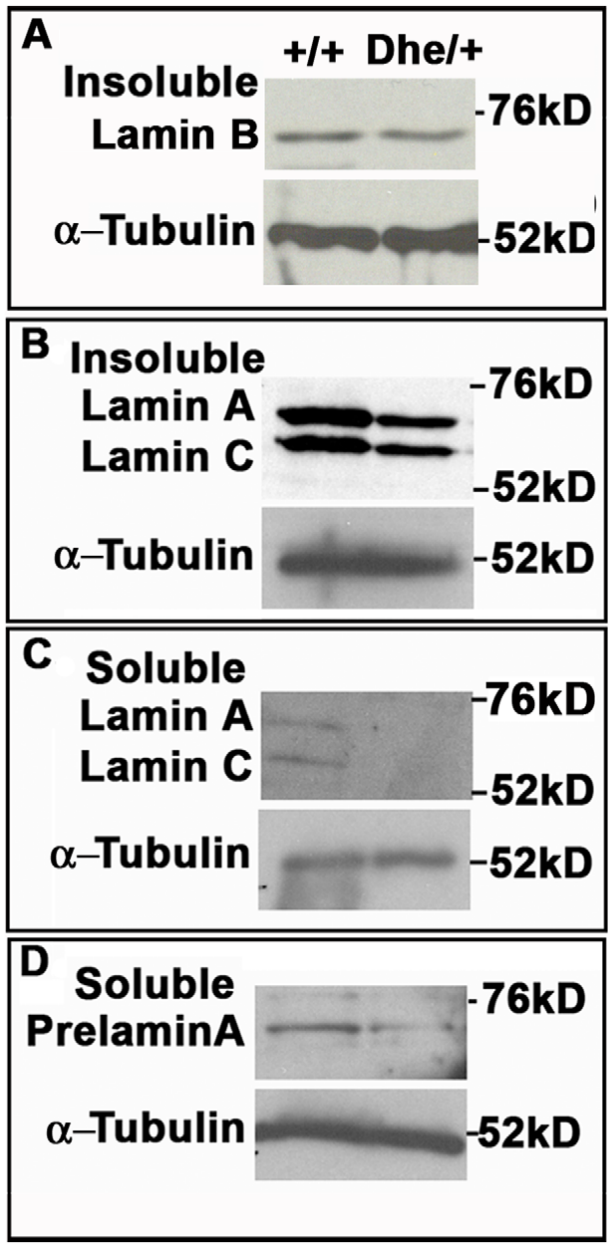
*Lmna^Dhe/+^* fibroblasts exhibit lower Lamin A levels, but no change in Lamin B expression. (A–D) Western blot analysis of insoluble Lamin B (A), insoluble Lamin A (B), soluble Lamin A (C) and soluble Prelamin A (D). These data indicate no difference in Lamin B protein levels between *Lmna^+/+^*and mutant cells, but less soluble Lamin A/C, insoluble Lamin A/C and Prelamin A protein in mutant cells. α-Tubulin was used as a loading control for all western blots.

We next tested whether the *Lmna^Dhe^* allele affected nuclear lamina structure. Cells labeled with anti-LMNA and -LMNB antibodies were imaged by 3D confocal microscopy. Confocal optical sections of the top and bottom of mutant nuclei revealed patches of irregularity in the normal, criss-cross pattern of the LMNA and LMNB meshworks (59.4+/−4% of cells; N = 100), unlike the regularly patterned network of lamins seen in *Lmna^+/+^*cells (2+/−2.8%; N = 84; p<0.001; χ^2^-test) (Figure 2 A–F). These “holes” in the meshworks were particularly noticeable in blebbed regions of mutant nuclei (Figure 2 B, D and F; white arrowheads). Western blotting indicated similar amounts of LMNB in normal and mutant fibroblasts (Figure 3A). Given the greater size of *Lmna^Dhe/+^* nuclei, these measurements suggest a lower density of LMNB in the mutant cell nuclei, consistent with the loss of protein from blebs (Figure 1D). Notably, both Prelamin A and LMNA were less abundant in both soluble and insoluble fractions in mutant cells, suggesting that LMNA is even less dense than LMNB in the lamina meshwork (Figure 3B, C and D).

**Figure 4:**
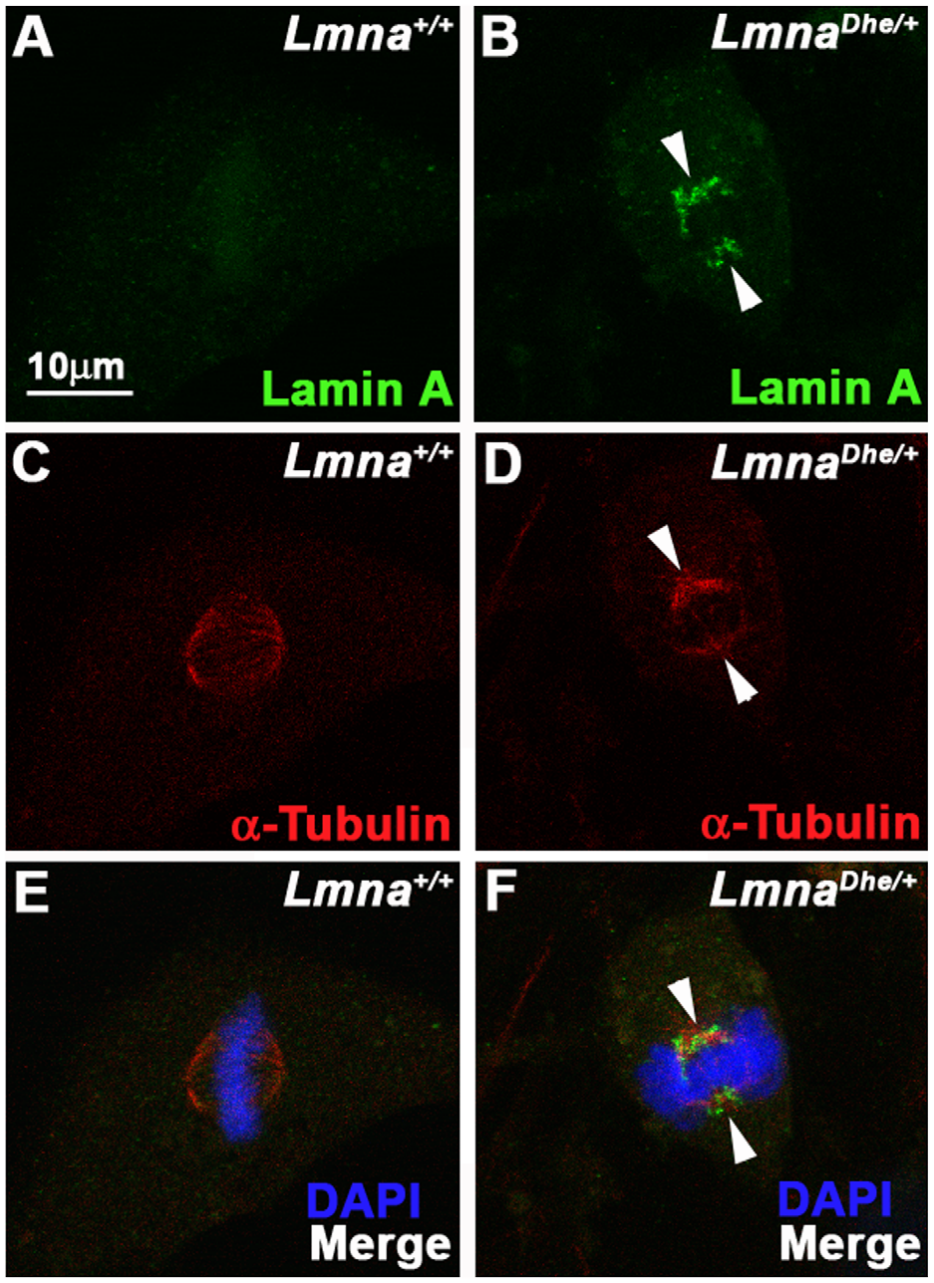
LMNA persists and colocalizes with the spindle apparatus during metaphase in *Lmna^Dhe/+^* cells. (A–F) Immunodetection of Lamin A (green;A,B,E,F) and α-Tubulin (red; C–F) in *Lmna^+/+^*(A,C,E) and *Lmna^Dhe/+^* (B,D,F) fibroblasts. Labeling indicates persistence of Lamin A in mitotic cells and colocalization with α-Tubulin during metaphase of mutant cells (white arrowheads), as opposed to *Lmna^+/+^*cells. Single confocal optical sections through the middle of the cell are shown. The metaphase chromosomes are counterstained with DAPI (blue: E, F).

Similar to human progeria cells, *Lmna^Dhe/+^* fibroblasts exhibited perturbations to LMNA disassembly during mitosis (Figure 4B, D and F) [6]. Specifically, residual LMNA aggregates clustered close to the spindle poles as well as throughout the mitotic spindle in metaphase in all observed mitotic cells. In contrast, LMNA in *Lmna^+/+^*cells was disassembled much earlier in mitosis and was nearly undetectable at metaphase (Figure 4A, C and E). Cytoplasmic aggregates were not detected in *Lmna^Dhe/+^* mitotic cells, in contrast to a previous report of fibroblasts transfected with *Lmna^HGPS^* [6]. LMNB disassembled normally during mitosis in *Lmna^Dhe/+^*cells (data not shown). Together, these data suggest that LMNA expression and assembly at the nuclear lamina is perturbed in *Lmna^Dhe/+^* cells during interphase and mitosis. In addition, *Lmna^Dhe^* influences the structure of the LMNB meshwork, but this influence is restricted to interphase.

### *Lmna^Dhe/+^* cells exhibit extensive aneuploidy

**Figure 5:**
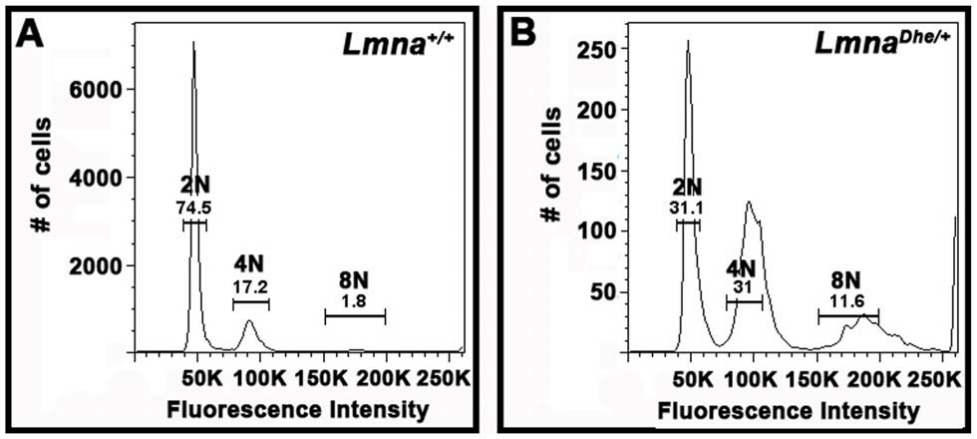
*Lmna^Dhe/+^* fibroblasts have increased DNA content subsequent to pervasive aneuploidy. (A and B) Flow cytometry analysis of propidium iodide labeling of *Lmna^+/+^*(A) and *Lmna^Dhe/+^* (B) dermal fibroblasts indicates that mutant cells have a significantly higher proportion of cells with 4C and 8C and lower fraction of 2C cells, as compared to *Lmna^+/+^*cell populations (p<0.01, Student's t-test). DNA content peaks for *Lmna^Dhe/+^* were also broader than *Lmna^+/+^*peaks, indicating a population of cells with a large range of DNA content.

The large nuclear size of *Lmna^Dhe/+^* fibroblasts suggested that the cells might be aneuploid, which is often a consequence of cell cycle defects [24], [39]–[41]. To test this we used flow cytometry to determine DNA content in each cell. We found a significantly higher fraction of cells that were greater than 4C in mutant as compared to *Lmna^+/+^*cultures. Likewise a much lower proportion of *Lmna^Dhe/+^* cells were 2C (p<0.01; χ^2^-test) (Figure 5A and B). In addition, the peaks surrounding 2C and 4C cells were broader in *Lmna^Dhe/+^* cells, suggesting a range of genome sizes.

**Figure 6:**
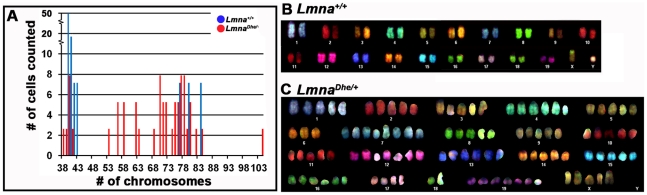
Spectral karyotyping reveals pervasive aneuploidy in *Lmna^Dhe/+^* fibroblasts. (A) Histogram of chromosome numbers in *Lmna^Dhe/+^* (red bars) and *Lmna^+/+^* (blue bars) dermal fibroblasts, as determined by metaphase spreading and SKY. Over 90% of mutant cells possessed an abnormal number of chromosomes ranging from 38 to 104. (B and C) Representative spectral karyotypes of *Lmna^+/+^* (B) and *Lmna^Dhe/+^* (C) cells. The wild-type cell exhibited a normal karyotype of 40XY (B), while the mutant cell was aneuploid with a 104XXXXX karyotype (C).

Increased DNA content, as observed above, suggested that *Lmna^Dhe/+^* cells might harbor chromosome abnormalities. Indeed, spectral karyotyping (SKY) indicated widespread aneuploidy in *Lmna^Dhe/+^* fibroblasts (92.5%) with chromosome numbers ranging from 38 to 104 chromosomes per nucleus (Figure 6A). In general, *Lmna^Dhe/+^* cells tended to be tetraploid, though chromosome copy numbers up to octoploidy were detected. In contrast, aneuploidy was detected in only 20% of *Lmna^+/+^*cells and typically involved gains or losses of a single chromosome. No specific chromosome was found to be consistently duplicated in mutant cells (Figure 6B and C). The wide variation in chromosome number in mutant cells is consistent with the large range of nuclear volumes observed (Figure 1G; Figure 5). The increased nuclear volume and subsequent extensive chromosome duplications suggested that the *Lmna^Dhe^* allele alters cell division in some way.

### Increased DNA damage in *Lmna^Dhe/+^* fibroblasts

The extensive nuclear blebbing in *Lmna^Dhe/+^* fibroblasts, as well as the pervasive aneuploidy, led us to examine whether the growth of these cells is affected in similar ways to other *Lmna* mutations [6]. We measured the growth rate of *Lmna^+/+^*and *Lmna^Dhe/+^* fibroblasts *in vitro* and found that *Lmna^+/+^*cells grew significantly faster than *Lmna^Dhe/+^* cells (p≤0.05, Student's t-test) (Table 1) [32], [33]. We then measured the proportion of senescent and actively cycling cells in *Lmna^Dhe/+^* cultures using senescence-associated β-galactosidase (SA-β-gal) and Ki-67 as markers, respectively. In contrast to *Lmna^HGPS^* and some other *Lmna* alleles, *Lmna^Dhe/+^* cells did not exhibit a significant proportion of cells positive for SA-β-gal as compared to *Lmna^+/+^* fibroblast cultures (p = 0.3; Student's t-test) (Table 1). *Lmna^Dhe/+^* cells also did not have a significantly different proportion of cells entering apoptosis, as determined by Annexin V labeling, an early marker of apoptosis (p = 0.9, Student's t-test; Table 1). However, fewer Ki-67 positive cells were found in *Lmna^Dhe/+^* cultures as compared to *Lmna^+/+^* cultures (p = 0.06, Student's t-test) (Table 1). These results indicated that a smaller fraction of *Lmna^Dhe/+^* fibroblasts was actively cycling, with no additional senescent cells or changes in apoptosis, as compared to *Lmna^+/+^* cultures.

**Table 1:** Cell cycling properties of *Lmna^Dhe/+^* fibroblasts.

	*Lmna^+/+^* 1	Number of cells	*Lmna^Dhe/+^* 1	Number of cells	p-value^7^
Growth^2^	270+/−55%	80,000	152+/−20%	80,000	0.02
Senescent^3^	18.9+/−3.3%	264,361	13.8+/−12%	179,948	0.3
Apoptotic^5^	15.3+/−2.1%	147,335	15.6+/−2.4%	85,482	0.9
Cycling^4^	18.6+/−5.8%	635	9.6+/−4.9%	1125	0.06
Mitotic^6^	1.1+/−0.9%	1879	2.3+/−0.1%	1089	0.07

1 Means and standard deviations for cultures from three mice are reported.

2 Culture growth over four days measured by static adhesion assay.

3 Proportion of all cells positive for SA-β-gal fluorescence versus total cells counted by flow cytometry.

4 Cycling cells identified by Ki-67 immunostaining.

5 Apoptosis measured by Annexin V labeling and flow cytometry.

6 Mitotic indices determined from cells stained with anti-Lamin A and anti-α-Tubulin antibodies and DAPI.

7 p-values were computed using Student's t-test.

**Figure 7:**
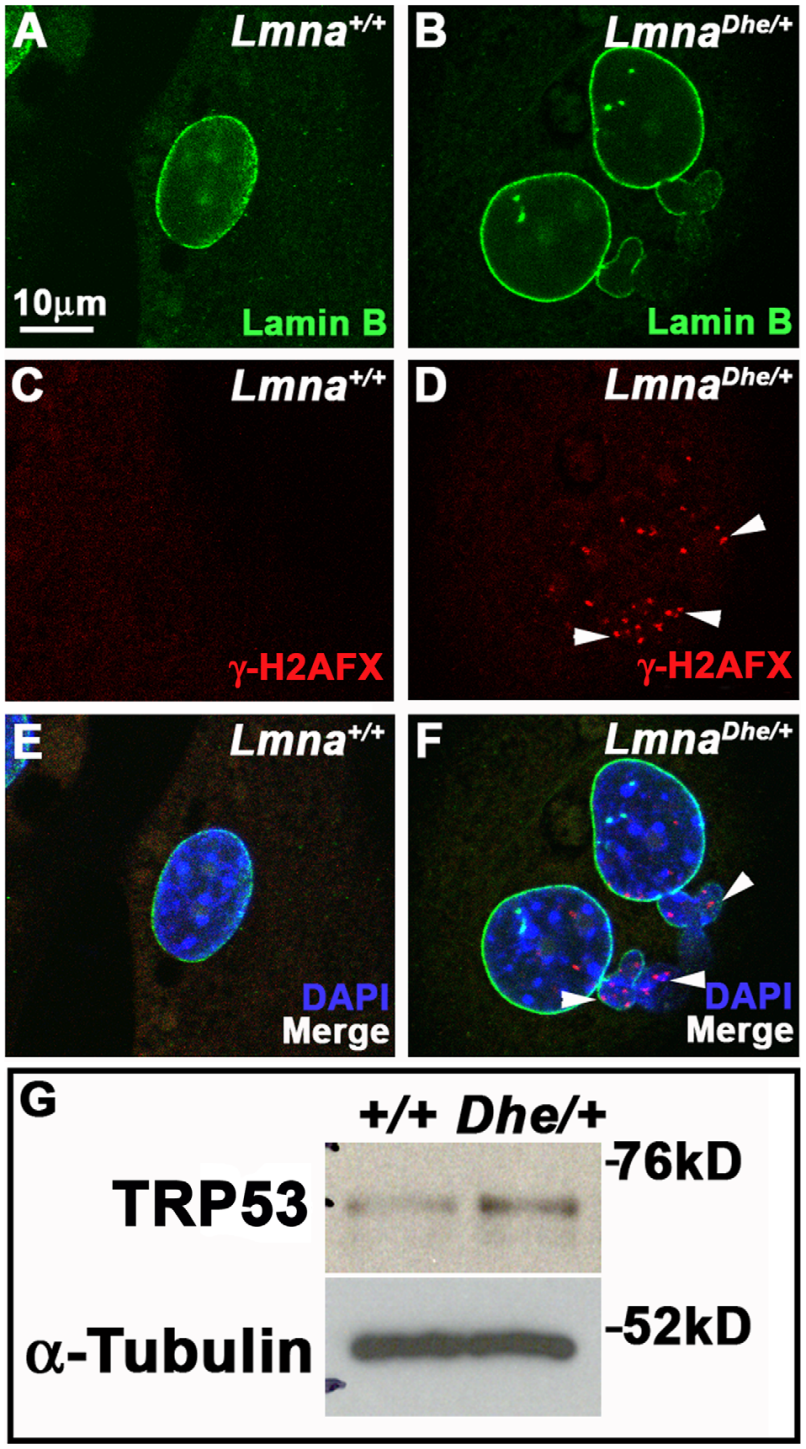
*Lmna^Dhe/+^* fibroblasts have foci of γH2AX and increased levels of TRP53 indicating DNA damage. (A–F) Immunodetection of Lamin B (green; A,B, E,F) and γH2AX (red; C–F) in *Lmna^+/+^*(A,C,E) and *Lmna^Dhe/+^* (B,D,F) fibroblasts. Mutant fibroblast nuclei have several foci of γH2AX throughout both the blebbed and non-blebbed areas of the nucleus (white arrowheads in D and F), in contrast to *Lmna^+/+^*nuclei. Single confocal optical sections though the middle of the nucleus are shown. Nuclei are counterstained with DAPI. (G) Western blots of total TRP53 in mutant and *Lmna^+/+^*fibroblasts show higher levels of TRP53 in mutant cells than in *Lmna^+/+^* cells. α-Tubulin was used as loading control.

The slow growth rate indicative of other *Lmna/LMNA* mutations is often accompanied by increased DNA damage [19]–[21]. Indeed, *Lmna^Dhe/+^* fibroblasts had numerous foci of the DNA damage marker, phosphorylated H2AFX (γH2AFX), present in both the main body of the nucleus as well as in blebbed regions, in marked contrast to *Lmna^+/+^* cells (Figure 7A–F). Western blotting indicated that *Lmna^Dhe/+^* cells also expressed higher levels of phosphorylated TRP53 then *Lmna^+/+^*cells (Figure 7G). These data suggest that although the apoptotic pathway is intact to the point of TRP53 activation, it is not promoting increased apoptosis in *Lmna^Dhe/+^* cells.

### Mitotic chromosome cohesion defects in *Lmna^Dhe/+^* fibroblasts

The extensive aneuploidy and DNA damage observed in *Lmna^Dhe/+^* cells led us to examine whether these cells also had mitotic defects, which could further decrease mutant cell growth rates. First, we determined the mitotic indices of asynchronous cultures of *Lmna^+/+^*and mutant fibroblasts. Interestingly, we noticed two-fold more *Lmna^Dhe/+^* mitotic cells as compared to *Lmna^+/+^*cells (p≤0.07, Student's t-test) (Table 1). Next, we tested whether *Lmna^Dhe/+^* fibroblasts arrested at a particular stage in mitosis. We found no significant difference in the proportions of prophase and prometaphase cells in *Lmna^+/+^*and *Lmna^Dhe/+^* cells. *Lmna^Dhe/+^* cultures had an increased proportion of cells in anaphase compare to normal cells (20% vs. 13%, p = 0.07, χ^2^-test; Table 2). Other stages of mitosis were not significantly affected (Table 2).

**Table 2:** Mitotic progression in *Lmna^+/+^* and *Lmna^Dhe/+^* cells.

Mitotic stage^1^	*Lmna^+/+^*	Number of cells^2^	*Lmna^Dhe/+^*	Number of cells^2^	p-value^3^
Prophase	26%	230	22%	174	0.5
Prometaphase	10%	230	13%	174	0.3
Metaphase	17%	230	14%	174	0.6
Anaphase	13%	230	20%	174	0.07
Telophase	35%	230	30%	174	0.4

1 Mitotic stages determined in fibroblasts immunofluorescently labeled with anti-Lamin A and anti-α-Tubulin antibodies and DAPI counterstain.

2 Total number of cells from two experiments.

3 p-values were computed using the χ^2^-test.

**Figure 8:**
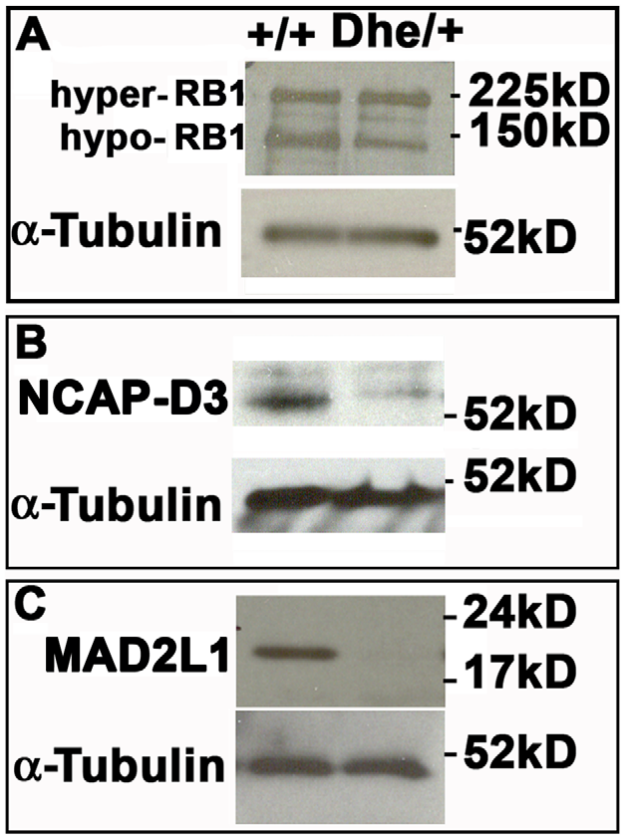
Decreased levels of hypophosphorylated RB1, NCAP-D3 and MAD2L1 in *Lmna^Dhe/+^* fibroblasts. (A) Western blotting indicated a lower level of activated, hypo-phosphorylated RB1 (150 kD band) in mutant cells, while hyper-phosphorylated RB1 levels were slightly elevated (225 kD band). (B) *Lmna^Dhe/+^* fibroblasts express lower levels of the centromere-specific cohesin subunit, NCAP-D3, as well. (C) *Lmna^Dhe/+^* cells express little to no detectable MAD2L1, a spindle checkpoint protein, while *Lmna^+/+^*cells robustly expressed this protein. α-Tubulin was used as a loading control for all western blots.

**Figure 9:**
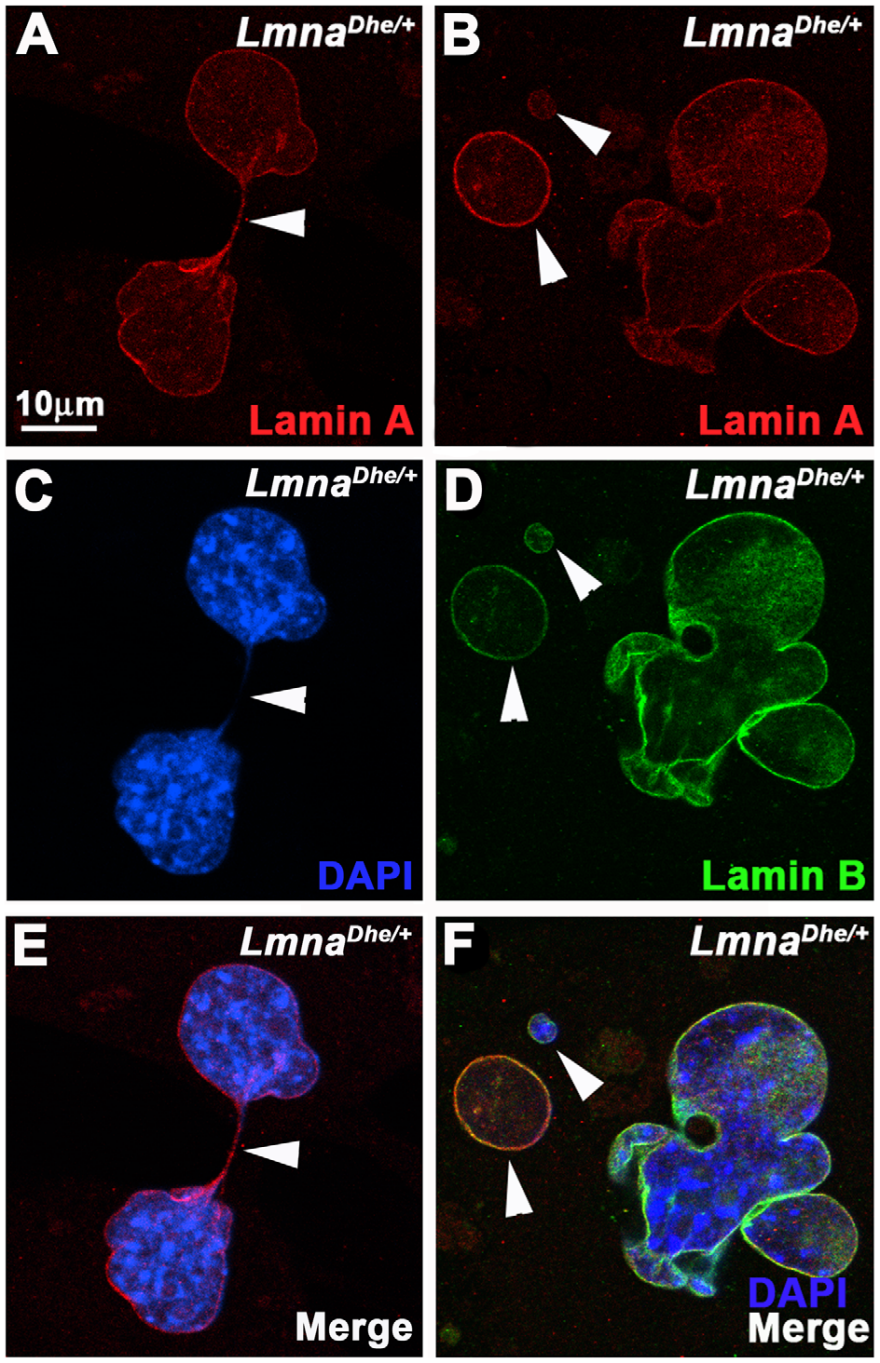
Increased occurrence of micronuclei and anaphase bridges in *Lmna^Dhe/+^* fibroblasts indicate segregation defects during mitosis. (A–F) Immunodetection of Lamin A (red; A,B,E,F) and Lamin B (green; C–F) in *Lmna^Dhe/+^* fibroblasts. Mutant fibroblast cultures have an increased proportion of cells with anaphase bridges (A,C,E; white arrowheads) (Table 3; p<0.001; t-test) and micronuclei (B,D,F; white arrowheads) (Table 3; p<0.001; t-test), as compared to *Lmna^+/+^* cells, consistent with chromosome segregation defects during mitosis. Single confocal optical sections though the middle of the nucleus are shown. Nuclei are counterstained with DAPI.

An anaphase delay in *Lmna^Dhe/+^* cells suggests chromosome segregation defects. Recent work has shown that decreased levels of RB1 caused centromere condensation and cohesion defects in anaphase due to decreased expression of NCAP-D3, the centromere-specific condensin II subunit [23], [26]–[28]. To test whether this pathway was perturbed in *Lmna^Dhe/+^* cells, we examined the levels of RB1 and NCAP-D3 by Western blotting. The levels of the active hypophosphorylated RB1 were lower in *Lmna^Dhe/+^* cells than those found in *Lmna^+/+^*cells (Figure 8A). *Lmna^Dhe/+^* fibroblasts also expressed lower levels of NCAP-D3 than *Lmna^+/+^*cells (Figure 8B), consistent with the decrease in hypophosphorylated RB1. We further determined whether a spindle checkpoint protein was present in *Lmna^Dhe/+^* fibroblasts. Immunoblotting indicated little to no detectable levels of mitotic arrest deficient-like 1 (MAD2L1), a spindle checkpoint protein that is degraded upon activation of APC/C^Cdc20^ after proper spindle attachment (Figure 8C). Reduced levels of NCAP-D3 and MAD2L1 may lead to chromosome segregation defects and aneuploidy in *Lmna^Dhe/+^* cells. Consistent with this, we observed a significant increase in the number of anaphase bridges formed as compared to *Lmna^+/+^*cells (p≤0.01, χ^2^-test; Figure 9A, C, E; Table 3). In addition, we noted an increase in the number of micronuclei in *Lmna^Dhe/+^* (11%+/−1.6) cells versus *Lmna^+/+^* (1.3%+/−1.5, p<0.001, χ^2^-test, Table 3; Figure 9B, D, F). These features suggest that reduced levels of NCAP-D3 and MAD2L1 are associated with aneuploidy in *Lmna^Dhe/+^* fibroblasts.

**Table 3:** Chromosome segregation defects in *Lmna^Dhe/+^* fibroblast cultures.

	*Lmna^+/+^* 2	Number of cells	*Lmna^Dhe/+^* 2	Number of cells	p-value^3^
Micronuclei^1^	1.25+/−1.5%	1500	11+/−1.6%	1155	≤0.05
Anaphase Bridges^1^	0.1+/−0.03%	1500	4.1+/−0.2%	1155	≤0.05

1 Micronuclei and anaphase bridges scored in cells immunofluorescently labeled with anti-LMNA and anti-Lamin B antibodies and DAPI counterstained.

2 Means and standard deviations for cultures from three mice are reported.

3 p-values were computed using the Student's t-test.

## Discussion

Using the *Lmna^Dhe^* mouse model, which recapitulates some physiological phenotypes of HGPS and other non-muscle involved laminopathies, we show here that the slowed cell growth caused by *Lmna* mutation is in part due to mitotic defects that include loss of the centromere-specific condensin subunit, NCAP-D3, and a defective spindle checkpoint. The mitotic defects are consistent with mis-regulation of RB1 in *Lmna^Dhe/+^* dermal fibroblasts and subsequent affects on sister chromatid condensation and cohesion through loss of NCAP-D3. Accordingly, the mitotically defective *Lmna^Dhe/+^* fibroblasts exhibited pervasive aneuploidy with high chromosome copy numbers. In addition, mitotic defects and increased DNA damage in interphase also were detected in *Lmna^Dhe/+^* cells. Thus, this study begins to fill in the missing link between the loss of RB1 activity in interphase and aberrant events in mitosis observed in other laminopathies.

### *Lmna^Dhe^* as a model for HGPS and related laminopathies

The *Lmna^Dhe^* mouse model studied here exhibits physiological phenotypes similar to human progeria and related laminopathies characterized by lipodystrophy, cranial abnormalities, and skin defects [29]. Here we show that the *Lmna^Dhe^* allele confers several of the cellular phenotypes observed in these diseases. These phenotypes include nuclear morphology defects, DNA damage, mitotic defects, and aneuploidy. Unlike HGPS patients, *Lmna^Dhe/+^* mice do not age prematurely (L.R. Donahue, unpublished). Consistent with their normal lifespan, cells from *Lmna^Dhe/+^* mice also did not senesce prematurely, although they did exhibit other cell cycle defects. Thus, *Lmna^Dhe^* may be a useful model for teasing apart mechanisms underlying the many cellular defects of HGPS and other non-muscle affected laminopathies. We specifically used this model to extend our understanding of mechanisms underlying cell cycle delay and aneuploidy in laminopathy cells.

### Mitotic defects in *Lmna^Dhe/+^* cells

Our data demonstrate that *Lmna^Dhe/+^* fibroblasts grow slower than *Lmna^+/+^*counterparts in part due to mitotic defects. Defects in mitosis have been reported previously for other LMNA mutant cells [6]. Here, we further show that *Lmna^Dhe/+^* mitotic defects were accompanied by low levels of NCAP-D3 and hypophosphorylated RB1. Recent reports indicate that these two proteins interact in primary cells and tumor cell lines, and that loss of RB1 activity subsequently perturbs chromosome condensation, sister chromatid cohesion and the spindle checkpoint. Failed interactions between RB1 and NCAP-D3 in primary and tumor cells therefore result in loss of chromosome segregation fidelity, aneuploidy, micronuclei and anaphase bridges, all of which were observed in *Lmna^Dhe/+^* cells as well [7], [13]–[15], [19]–[22], [24]. Whereas the low levels of hypophosphorylated RB1 reported for other *Lmna*/*LMNA* deficient cells have been implicated in altering the G1/S transition, our findings with *Lmna^Dhe/+^* fibroblasts suggest that the influence of RB1 extends to mitosis as well [11], [19], [26], [28], [39]–[42].

Consistent with our findings in *Lmna^Dhe/+^* fibroblasts, gene expression data implicate a loss of the RB1 pathway in HGPS patient cells [16], [17]. Furthermore, aneuploidy has been detected in a variety of HGPS and related laminopathies as well [22], [24]. For example, a mouse model of HGPS that is deficient for ZMPSTE24 exhibits aneuploidy in up to 40% of cultured cells [22]. In human premature aging syndromes, up to 30% of patient cells exhibit aneuploidy [21], [22], [24]. While aneuploidy in itself is not a novel phenotype for *LMNA^HGPS^* and related alleles, the extent of aneuploidy present in *Lmna^Dhe/+^* cells is unique. Aneuploidy may be further exacerbated in *Lmna^Dhe/+^* cells by loss of proper spindle checkpoint proteins, such as MAD2L1.

### DNA damage in *Lmna^Dhe/+^* fibroblasts

The high level of aneuploidy in *Lmna^Dhe/+^* cells predicts that these mice might be predisposed to developing tumors, but they are not. Instead, *Lmna^Dhe/+^* cells grew slowly. They were not prone to premature senescence, indicating that other mechanisms of cell growth control were defective in *Lmna^Dhe^* cells. Senescence in HGPS is linked to increased levels of DNA damage, also observed in *Lmna^Dhe/+^* cells. Thus, premature senescence and DNA damage are uncoupled in *Lmna^Dhe/+^* cells. At least three known mechanisms underlie LMNA-dependent DNA damage: 1) Mislocalization of the replication factors, proliferating cell nuclear antigen (PCNA) and polymerase δ, resulting in stalled replication forks [15], [20], [22], 2) defective DNA damage repair pathways [7], [15], [20], [22], [24], and 3) telomere dysfunction [18]. In *Lmna^Dhe/+^* fibroblasts and other *Lmna/LMNA* mutant cells, the TRP53 pathway is activated subsequent to DNA damage, although further downstream activation of apoptosis fails [20]–[22], [43]. Increased DNA damage in *Lmna*/*LMNA* mutants might contribute to additional defects in mitosis, since unresolved DNA damage and aberrant DNA repair could manifest as increased levels of aneuploidy, lagging chromosomes, anaphase bridges and micronuclei [7], [19], [20], [22], [24], [39]–[42].

### Independent, but interconnected networks at the nuclear lamina

LMNA and LMNB have been described as independent, but interconnected networks of proteins comprising the nuclear lamina [4]. We show here that the *Lmna^Dhe^* allele had profound effects on both the LMNA and LMNB networks. LMNA expression was curtailed in *Lmna^Dhe/+^* fibroblasts, resulting in an overall lower density of LMNA throughout the nucleus. Reduction in LMNB density also was detected, but to a lesser extent. In contrast to the dominant mutation described here, siRNA knockdown of LMNA does not affect the LMNB network in HeLa cells [4]. However, knockdown of LMNB1 induces large holes in the LMNA and LMNB2 network, supporting interdependency of the networks [4]. Furthermore, this interdependency may be cell cycle stage specific. Our data suggest that LMNA can influence LMNB in interphase, but that the normal dissolution of LMNB in mitosis is unaffected by *Lmna^Dhe^*, which persists in mitosis.

In addition to decreases in the overall density of LMNA and LMNB in *Lmna^Dhe/+^* fibroblasts, perturbations to nuclear lamina structure also were detected. Specifically, the LMNB distribution was skewed toward depletion from the majority of nuclear blebs. This redistribution was not found for LMNA. Furthermore, both LMNA and the residual LMNB networks exhibited large, irregular holes in blebs, indicating aberrant assembly of lamin filaments in these regions. We envision two ways in which these networks were perturbed: (1) LMNA^Dhe^ might have influenced the nuclear membrane to form blebs, which then indirectly pulled the lamina into a less dense network in these regions, or (2) LMNA^Dhe^ directly caused the perturbation of both LMNA and LMNB networks. *Lmna^Dhe^* is caused by a dominant mutation in a critical leucine of the first coiled coil domain, which is involved in filament formation. Thus, *Lmna^Dhe^* might form aberrant, irregular filaments in the nucleus. These possibilities are not necessarily mutually exclusive and await testing in future studies.
